# Coexistence of Hodgkin Lymphoma and Squamous Cell Carcinoma of the Tongue: A Case Report and Literature Review

**DOI:** 10.7759/cureus.75172

**Published:** 2024-12-05

**Authors:** Abdullah Alhajlah, Khaled A Almanea, Rawan M Alahmadi, Saeed M Alqahtani, Saad Algahtani, Fareed R Alghamdi, Nagoud Ali

**Affiliations:** 1 College of Medicine, Imam Mohammad Ibn Saud Islamic University, Riyadh, SAU; 2 Otolaryngology - Head and Neck Surgery, Prince Sultan Military Medical City, Riyadh, SAU; 3 Pathology, Prince Sultan Military Medical City, Riyadh, SAU

**Keywords:** hodgkin lymphoma, multiple primary, oropharynx, secondary malignancy, squamous cell carcinoma, tongue neoplasm

## Abstract

We report an unusual case of a 39-year-old male patient with a previous history of treated classical Hodgkin lymphoma, presenting with tongue ulcer and left ear pain who was subsequently diagnosed with invasive squamous cell carcinoma of the oropharynx. This case highlights the importance of vigilance in patients with a history of lymphoma and the potential for the development of secondary malignancies. We discuss the clinical, radiological, and pathological findings and emphasize the need for close monitoring and early intervention in such cases.

## Introduction

The increased incidence of second malignant neoplasms (SMNs) after Hodgkin lymphoma (HL) has been well-documented in the literature [1-3]. In the head and neck region, surgeons encountering simultaneous primary malignancies like HL and squamous cell carcinoma (SCC) of the tongue are exceedingly rare, presenting unique diagnostic and treatment challenges [1-4]. This case, a notable addition to the limited literature, underscores the intricacy of managing such dual malignancies in the head and neck region, emphasizing the importance of a comprehensive, multidisciplinary approach to these complex clinical scenarios.

## Case presentation

A 39-year-old smoker with a history of classical HL of the right cervical lymph node, clinically stage 1A, diagnosed in 2017 and, previously treated with chemotherapy, lost follow-up for six years and presented with left tongue ulcer, an enlarged and tender left level 2 lymph node, and a persistent left ear pain.

Physical examination revealed a left lateral posterior tongue lesion and an enlarged, tender left level 2 lymph node. Further examination with a flexible scope showed a nasopharyngeal mass and the vocal cords were bilaterally movable with no masses. Otoscopy demonstrated a clear and intact tympanic membrane. Biopsies showed the existence of invasive, moderately differentiated SCC in the left lateral posterior tongue lesion, nonetheless, there was no evidence of malignancy infiltration in the nasopharynx. The imaging studies provided a comprehensive overview of the patient's condition.

**Figure 1:**
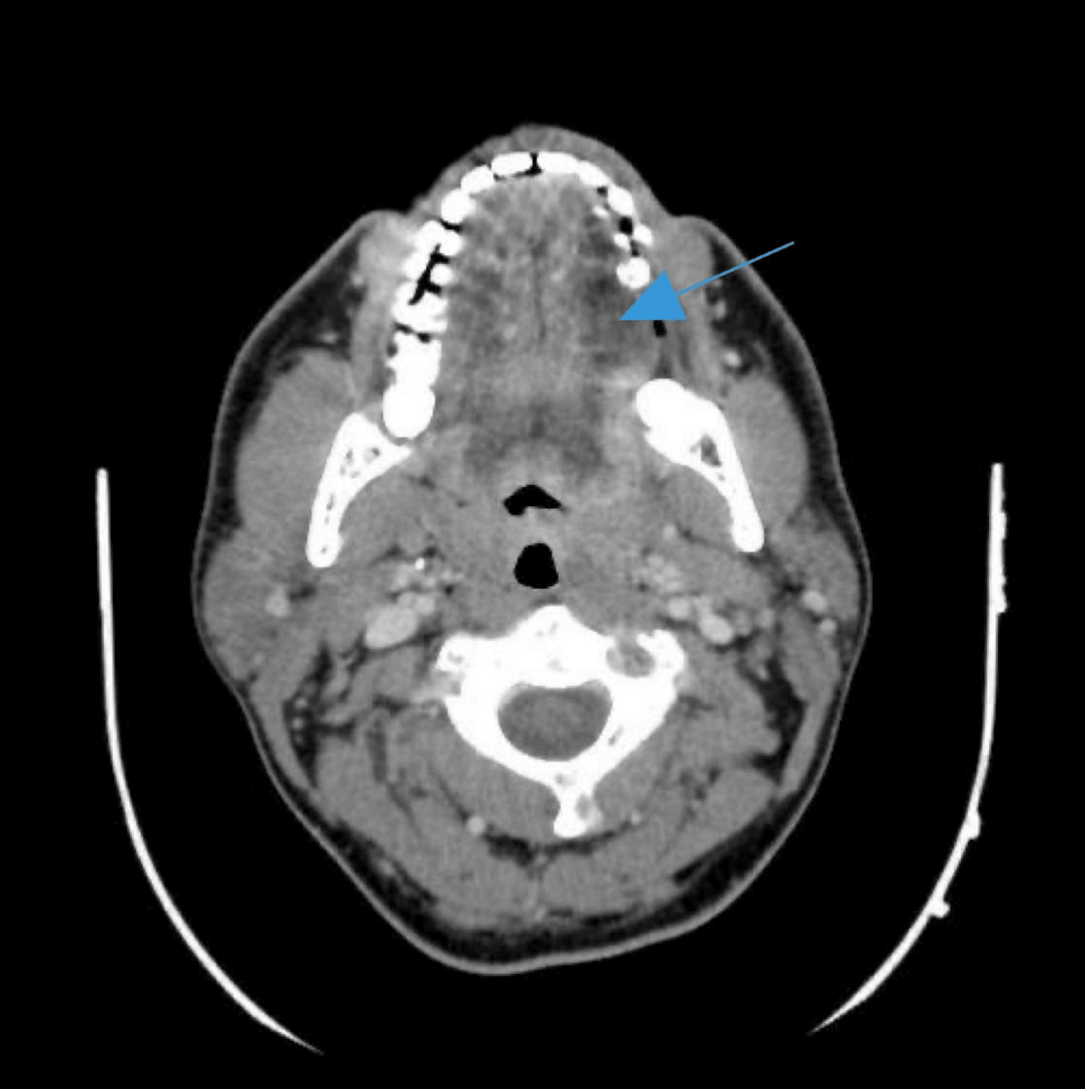
Axial computed tomography (CT) scan with contrast in the neck, demonstrating a left tongue hypodense lesion (blue arrow).

**Figure 2:**
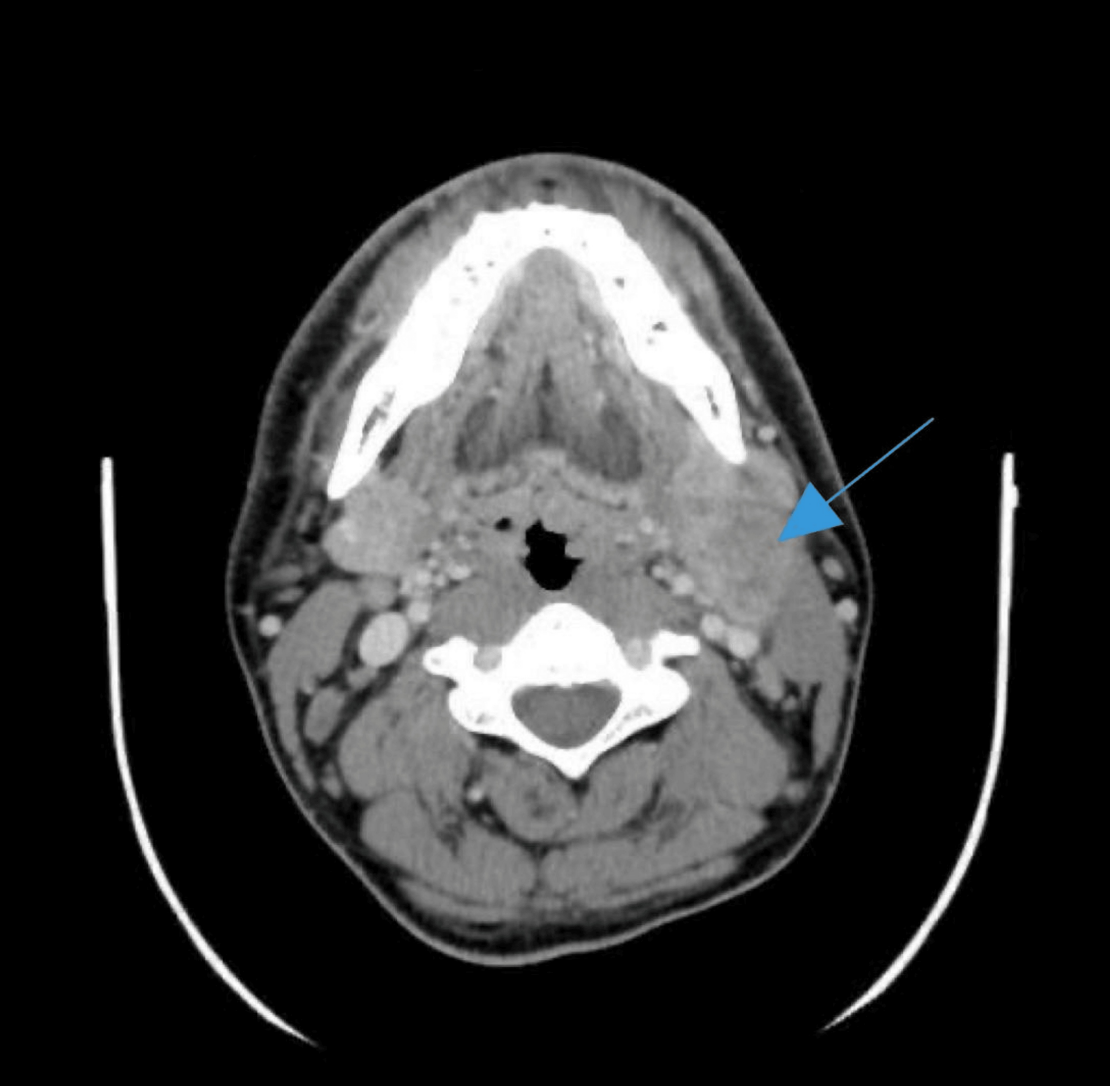
Axial computed tomography (CT) scan with contrast in the neck, demonstrating left level 2 lymph nodes with heterogenosity and central necrosis (blue arrow).

The CT scan of the head and neck showed increased fullness in the nasopharynx and mixed responses in the jugulodigastric lymphadenopathy, suggestive of disease progression (Figures 1-2).

**Figure 3:**
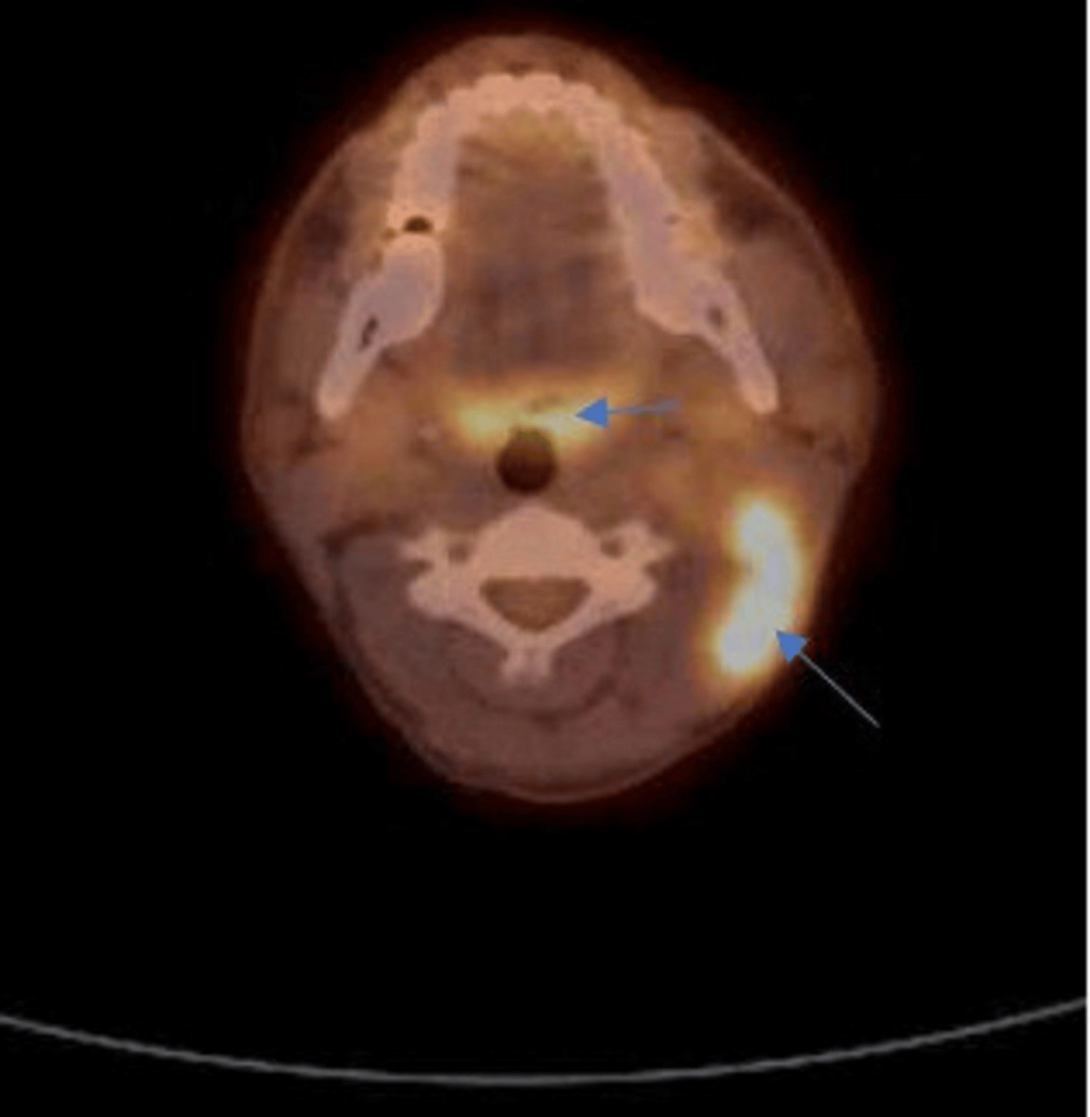
FDG PET-CT of the neck demonstrating hypermetabolic activity in the right upper cervical lymph node and the pre-epiglottic area (blue arrows). FDG PET-CT: fluorodeoxyglucose positron emission tomography-computed tomography

The PET-CT scan displayed prominent hypermetabolic activity in the oropharynx, specifically in the pre-epiglottic region and bilateral palatine tonsils, with significant metabolic activity in the left upper cervical lymph node, raising concerns for metastasis (Figure 3).

**Figure 4:**
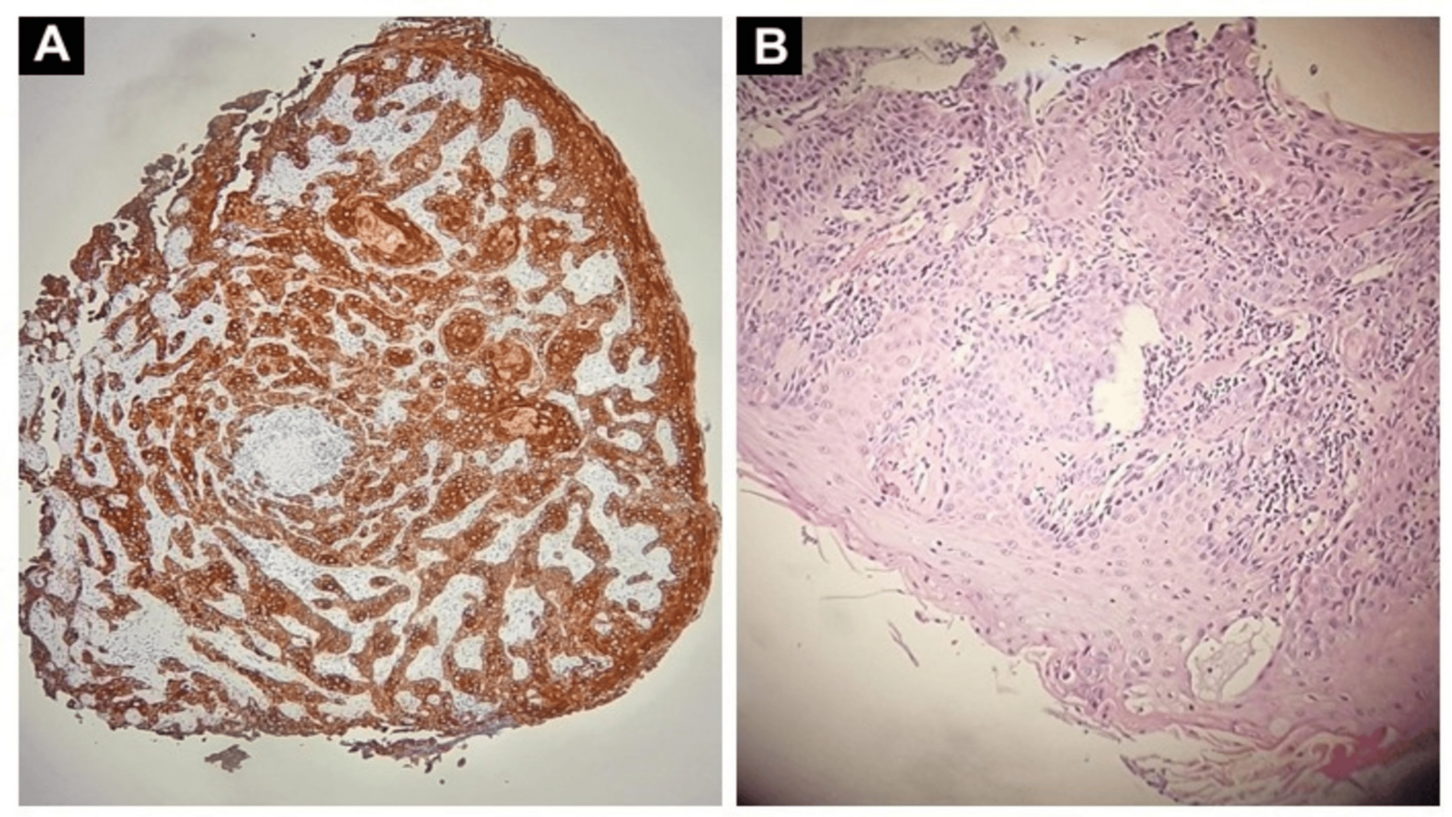
Immunohistochemistry showing Cytokeratin 5/6 positivity (A) and well-differentiated invasive squamous cell carcinoma in the background of inflammation in the tongue (B).

**Figure 5:**
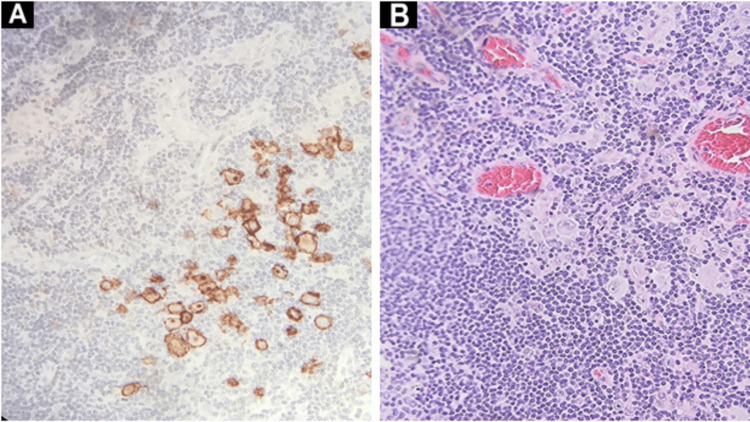
Immunohistochemistry showing CD30 positivity in Hodgkin lymphoma (A), and H&E-stained tissue of the cervical lymph node depicting the usual morphology for Reed-Sternberg (RS) cells (B).

These imaging findings, collectively, indicated a complex scenario with both progressive and regressive elements in different areas. The notable hypermetabolic activity and lymph node involvement initially aimed towards metastasis from the SCC of the tongue. A left partial glossectomy, bilateral tonsillectomy, and left radical neck dissection with nasopharyngeal biopsy were determined following a discussion of the case on the tumor board. However, the intraoperative pathology revealed a dual pathology. Contrary to the metastatic expectations from the tongue SCC (Figure 4), the lymph nodes were predominantly affected by HL (Figure 5).

Responded very well to the surgical approach, three months postoperatively, CT scans demonstrated negative findings and the patient regularly followed up every three months in our clinic. This unanticipated coexistence of SCC and HL within the lymph nodes significantly altered the clinical understanding and necessitated a revision in the treatment approach and prognosis.

## Discussion

HL predominantly manifests in lymphatic tissues but can occasionally present in extra-nodal sites, including the head and neck region [1-4]. HL is commonly seen in late adulthood, the occurrence alongside SCC of the tongue, as presented in this case report, highlights a rare and intricate clinical scenario [3,5]. The coexistence of these two distinct malignancies is not frequently documented, aligning with the literature that states multiple malignancies account for a small percentage (2-11%) of all head and neck malignancies​​​​ [4,5].

The increased occurrence of subsequent malignancies in HL survivors, as compared to the general populace, is partially a consequence of the long-term carcinogenic effects of chemotherapy and radiotherapy treatments. Furthermore, individuals who have overcome HL may possess a heightened predisposition towards the emergence of SMNs relative to survivors of other malignancies, a susceptibility that could be attributed to genetic factors inherent to HL [2,6-9].

SCC of the tongue is a highly prevalent malignancy that is more common in the head and neck regions. However, the simultaneous development of HL and tongue carcinoma, which is similar to the infrequently documented cases of laryngeal cancer and lymphoma occurring simultaneously, adds complexity to the diagnostic, therapeutic, and prognostication processes [1]. Similar to the case reported by Nigri and Khasgiwala where a patient with laryngeal SCC was later found to have HL, a synchronous lymphoma with laryngeal carcinoma in situ and a mucosa-associated lymphoid tissue (MALT)-type lymphoma with SCC of the larynx were reported as well [10,11]. Our case underscores the importance of a thorough histopathological examination in cases of suspected or known malignancies in the head and neck region​.

In the context of extra-laryngeal carcinoma coexisting with lymphoma, our case parallels the complexity seen in other reported cases where patients presented with multiple primary malignancies involving different regions of the head and neck. The development of a second malignancy in the presence of HL, while not as common as with well-differentiated lymphocytic lymphomas, remains a significant clinical consideration, especially in the head and neck region where lymphatic and mucosal tissues interact closely [12,13]. While non-Hodgkin lymphoma (NHL) has a noted association with human immunodeficiency virus (HIV)-infected patients, this association is less clear with HL [14].

Our patient's clinical presentation, without HIV infection, aligns with the typical demographic profile for HL. The decision-making process in such cases is nuanced and requires a balance between aggressive treatment for local control and the careful management of potential systemic disease spread [1,14]. Predisposing factors for the development of SCC, including chronic illness, smoking, and prior chemotherapy or radiotherapy, were considered in the management of our patient [6-8]. However, our case featured the synchronous development of both tumors without a history of the common predisposing factors, presenting a unique clinical picture.

The diagnosis and management of our patient highlighted the necessity for comprehensive and thorough pre-operative examinations to ensure all potential primary sites and regions of tumor involvement are adequately assessed. This case contributes to the growing body of literature on the rare but clinically significant occurrence of concurrent HL and SCC of the tongue, emphasizing the need for meticulous diagnostic workup and individualized, multidisciplinary treatment approaches in managing such complex clinical scenarios​​​.

## Conclusions

We present an extremely rare case of a patient with classical HL who subsequently developed invasive SCC of the tongue. This case highlights the importance of ongoing surveillance of cancer survivors and the potential for the development of secondary malignancies. A multidisciplinary approach is crucial for the diagnosis, treatment, and follow-up of patients with coexisting primary diseases.
